# Evaluation of Pharmacy Intern Interventions on Antimicrobial Use in University-Affiliated Hospitals: A Retrospective Analysis

**DOI:** 10.3390/jcm13175060

**Published:** 2024-08-26

**Authors:** Tahani M. Almeleebia, Khalid M. Orayj, Wael A. Alghamdi, Mona A. Almanasef, Omar Hany, Ahmed R. N. Ibrahim

**Affiliations:** 1 Department of Clinical Pharmacy, College of Pharmacy, King Khalid University, Abha 62521, Saudi Arabia; korayg@kku.edu.sa (K.M.O.); malmanasaef@kku.edu.sa (M.A.A.); oabdelmoaty@kku.edu.sa (O.H.); a.ibrahim@mu.edu.eg (A.R.N.I.); 2 King Khalid University Medical City, Abha 62223, Saudi Arabia; 3 Department of Biochemistry, Faculty of Pharmacy, Minia University, Minia 61111, Egypt

**Keywords:** antimicrobial stewardship, clinical pharmacy, pharmacy interns, interventions, antimicrobials, Saudi Arabia

## Abstract

**Background**: Appropriate use of antimicrobials is essential to enhance therapeutic safety and efficacy. Clinical pharmacists play a crucial role in optimizing antimicrobial use; however, the contribution of pharmacy interns in antimicrobial use has not been studied. The objective of this study was to ascertain the quantity and nature of interventions related to antimicrobials documented by pharmacy interns, along with the rates at which physicians accepted these interventions. **Methods**: From August 2017 to March 2022, this study retrospectively evaluated antimicrobial-related interventions recorded by pharmacy interns during their rotations at King Khalid University. The categories of interventions included medication selection, addition of antimicrobials, dose or frequency adjustments, medication discontinuation, de-escalation, therapeutic drug monitoring, and others. Statistical analysis was conducted to identify patterns and correlations. **Results**: This study evaluated 1295 antimicrobial-related interventions, with high physician acceptance rates of 91.6% and 4.0% accepted with modifications. The most frequent interventions were dose/frequency adjustments (36.3%) and medication discontinuation (23%). Vancomycin, colistin, and meropenem were the most frequently intervened antimicrobials. Documented clinical outcomes included enhancing treatment efficacy (37.3%), reducing treatment toxicity (26.81%), and avoiding unnecessary antimicrobial exposure (21.8%). Significant correlations were observed between hospital units and intervention types, indicating unit-specific intervention patterns. **Conclusions**: Theses findings highlight the vital role of pharmacy interns in optimizing antimicrobial therapy. Future research should focus on evaluating the long-term clinical and economic benefits of their involvement.

## 1. Introduction

Optimizing the use of antimicrobial drugs is crucial for achieving their intended efficacy and reducing adverse events. Irrational use of antimicrobials is one of the main drivers that contributes to accelerating drug resistance [1]. Drug-resistant infections pose significant consequences at the individual patient level and on a broader public health scale, often resulting in severe illnesses with limited treatment options and substantially elevating the risk of complications and mortality [2]. Antimicrobial resistance (AMR) leads to treatment failure, prolonged disease duration and hospitalization, escalated medical costs, and increased mortality [1,3]. A previous study estimated that in 2019, 1.27 million deaths were directly attributable to bacterial AMR, demonstrating a critical global health impact [4].

The World Health Organization highlights that AMR not only jeopardizes our ability to treat common infectious diseases but also compromises surgical outcomes and cancer chemotherapy, which relies on effective antimicrobial prophylaxis [5]. These findings highlight the urgent need to optimize and sustain antimicrobial use to curb the rise of drug-resistant pathogens, a concern exacerbated by declining investment in the development of new antimicrobials [5].

Implementing interventions of antimicrobial stewardship programs (ASPs) has been shown to have positive outcomes such as increasing policy compliance, reducing treatment duration, and improving clinical as well as economic outcomes [6,7]. Several studies have emphasized the significant role of pharmacist interventions in optimizing the use of antimicrobials and improving patient care. These pharmacist interventions are impactful regardless of formal ASP involvement [8,9,10,11]. Pharmacist roles may include developing and constantly updating antimicrobial policies and guidelines, reviewing patient charts with the purpose of optimizing therapy, managing antimicrobial formulary, and providing education and training to healthcare staff on the proper use of antimicrobials [12]. In 59% of hospital ASPs in the United States, pharmacists co-led the program with physicians according to the 2019 NHSN (National Healthcare Safety Network) annual hospital survey [13].

**Figure 1:**
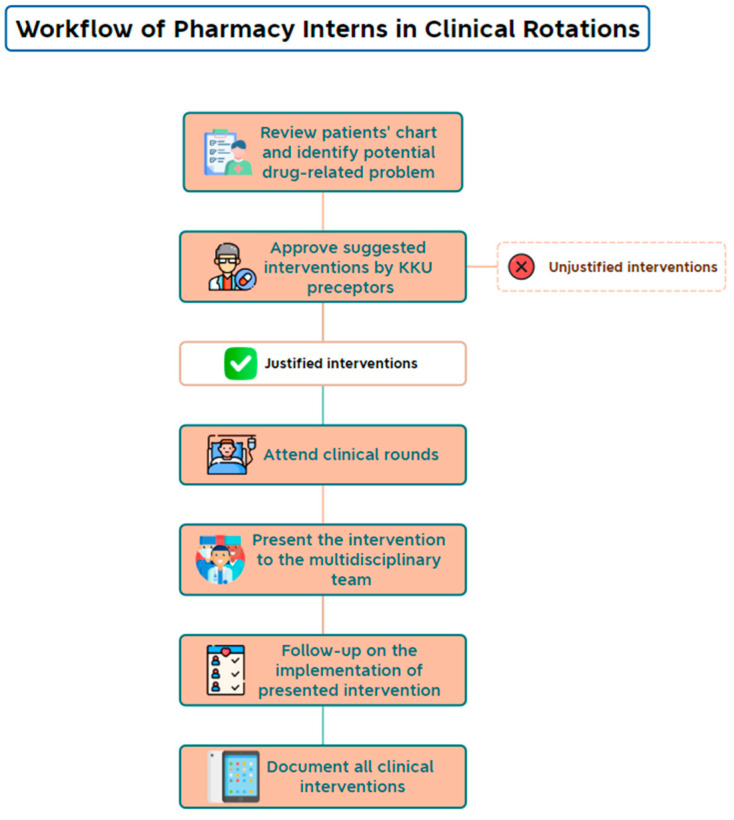
Workflow of pharmacy interns in clinical rotations.

The College of Pharmacy at King Khalid University (KKU) took the initiative to supplement the ongoing shortages of clinical pharmacists in the Asir region of Saudi Arabia by providing clinical pharmacy services at affiliated healthcare institutions. Pharmacy interns who were assigned to clinical rotation with faculty preceptors were required to review patient medical files for the proper use of medications under the supervision of clinical preceptors and document their interventions using a unified electronic form as part of their Advanced Pharmacy Practice Experiences (APPEs) (Figure 1). Pharmacy interns are undergraduate students who have begun their 1-year professional training after completing 5 years of didactic courses at a college of pharmacy. The aim of this study was to determine the number and type of antimicrobial-related interventions recorded by pharmacy interns and the acceptance rates of these interventions by physicians.

## 2. Materials and Methods

### 2.1. Study Design and Setting

A retrospective review of interventions documented by pharmacy interns and approved by KKU clinical preceptors at KKU-affiliated healthcare institutions was conducted to include all antimicrobial-related interventions from August 2017 to March 2022. The names of healthcare institutions will remain anonymous to minimize potential biases or stigmas. Clinical preceptors practice at five KKU-affiliated healthcare institutions: two specialized hospitals targeting two or fewer patient populations (A, B), and three general hospitals (C, D, E). The number of clinical preceptors varies among healthcare institutions, with eight clinical preceptors in Hospital C and one clinical preceptor in each of the other hospitals. Hospital B focuses on providing specialized healthcare and services tailored to the unique needs of a particular patient population, and managing infectious diseases is not the primary scope of practice.

### 2.2. Data Collection

Only the data captured by pharmacy interns were available for evaluation. Data extracted for each intervention included rotation types, healthcare institution names, clinical significance, expected outcomes, physician responses, and other intervention details. The interventions were then reviewed, and those related to antimicrobial use were included in the analysis. Interventions not related to antimicrobials and those involving vaccines were excluded from the study.

The extracted interventions were categorized as follows: addition of an antimicrobial, selection of an antimicrobial agent, discontinuation of an antimicrobial agent, switching from intravenous to oral (IV to PO) antimicrobials, drug information provision, dose or frequency adjustment, monitoring lab parameters, therapeutic drug monitoring (TDM), therapeutic consult, and antimicrobial de-escalation. De-escalation was defined as reducing the spectrum of antimicrobial therapy to a more targeted antimicrobial after initial broad-spectrum coverage, while discontinuation was defined as completely stopping an antimicrobial due to infection resolution or ongoing treatment determined to be unnecessary. Therapeutic consult involves a comprehensive assessment of the patient’s medical conditions, medication administration records, and microbiological data to request additional diagnostic measures or resolve drug-related problems such as interactions with other non-antimicrobial agents. Drug information provision addresses all general antimicrobial-related queries without assessing the patient. The interventions were also categorized based on their clinical significance to one of the following: avoiding unnecessary exposure to antimicrobial therapy, increasing the efficacy of therapy, reducing adverse effects/toxicities related to antimicrobial therapy, or unknown significance.

Antimicrobial agents are classified as antibacterial, antiviral, antifungal, antimalarial, or anthelmintic agents. Subclassification is also performed based on chemical and pharmacological classes. The “multiple classes” category was assigned to interventions that included a combined regimen from more than one class. Interventions that did not specify the drug by name were classified as “unspecified”.

### 2.3. Statistical Analysis

Frequencies and percentages were used to present the data. Crosstab analysis was used to identify differences in categorical variables. The Bonferroni–Holmes correction was used to keep a familywise error rate at 0.05 when there were multiple comparisons. When examining the relationship between clinical interventions and hospital units, the clinical intervention options were regrouped and renamed as follows: (1): “addition of an antimicrobial” and “selection of medication” were clumped into “select or add medication”; (2): “discontinuation of a medication” and “antimicrobial de-escalation” were clumped into “discontinue or de-escalate medication”; (3): “therapeutic consult” and “drug information” were clumped into “drug information”; and (4): “switching from IV to PO”, “TDM”, “dose or frequency adjustments”, and “monitoring lab parameter” were clumped into “dose modification and monitoring”. All statistical tests were two-sided. Differences were deemed statistically significant if the null hypothesis is rejected with greater than 95% certainty (*p* = 0.05).

### 2.4. Ethical Considerations

This study was ethically cleared by the Research Ethics Committee of King Khalid University (ECM#2022-1905). All interventions made by pharmacy interns and approved by preceptors were documented for quality purposes and no personal identifiable data were collected.

## 3. Results

Between 2017 and 2022, 3498 clinical pharmacy interventions were reported by pharmacy interns. Out of the 3498 interventions, 1295 were related to antimicrobial use. During the study period, a total of 328 pharmacy interns had at least one 5-week clinical rotation supervised by a KKU-clinical preceptor during their internship year. The proportion of pharmacy interns who made at least one intervention related to antimicrobial use is 81% (266 pharmacy interns), with an average of five interventions performed by a pharmacy intern. The majority of interventions occurred in general hospital D (66.0%) and specialized hospital A (25.7%). The highest proportion of interventions were recorded in the infectious disease unit (ID) (46.6%), followed by the pediatric intensive care unit (PICU) (25.7%), adult intensive care unit (ICU) (19.1%), and internal medicine (7.4%). Dose or frequency adjustment was the most common intervention (36.3%), followed by discontinuation of a medication (23%) and therapeutic consult (12.3%) (Table 1). More than 85% of the recorded interventions were carried out to increase the efficacy of antimicrobial therapy (37.3%), reduce adverse effects and toxicities (26.8%), or avoid unnecessary exposure to antimicrobial therapy (21.8%). Physicians accepted the majority of these interventions without modifications (91.6%) or with modifications (4.0%) (Table 1).

**Figure 2:**
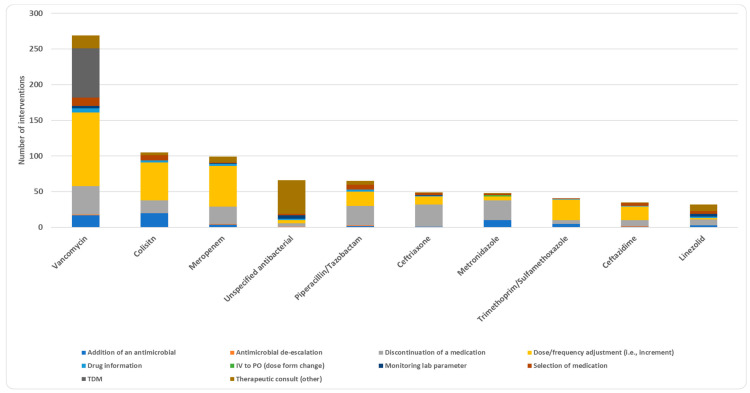
Type of interventions for the top 10 drugs where the interventions were applied.

**Figure 3:**
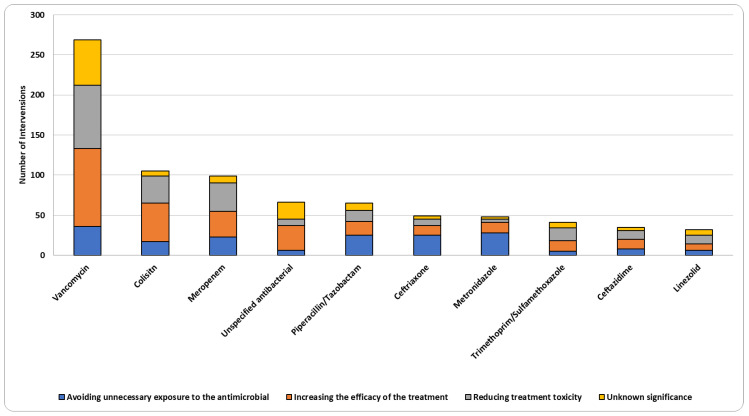
Clinical significance of interventions on the top 10 drugs.

Antibacterial agents were the most frequently used drug class (87.3%), followed by antifungals (5.9%) and antivirals (4.9%) (Table 2). Dose or frequency adjustments and discontinuation of medications were the most common interventions among antibacterial agents, with 37.1% and 23.8%, respectively (Supplementary Table S1). Vancomycin had the highest number of interventions, with a percentage of 20.8%, followed by colistin (8.1%) and meropenem (7.6%) (Supplementary Table S4). Dose or frequency adjustment intervention was the most prevalent among vancomycin interventions (38.3%), colistin (50.5%), and meropenem (57.6%) (Figure 2). Therapeutic drug monitoring (TDM) intervention was mainly applied to vancomycin (25.7%) out of all vancomycin interventions (Figure 2). When it comes to drug subclasses and among antibacterial agents (*n* = 1130), more than half of the interventions were related to glycopeptides (23.4%), cephalosporins (13.6%), carbapenems (11.2%), and polypeptides (9.3%) (Supplementary Tables S2 and S3). At medication level, the majority of vancomycin interventions were aimed at increasing the efficacy of vancomycin therapy (36.1%) and reducing adverse effects and toxicities of vancomycin (29.4%) (Figure 3).

**Figure 4:**
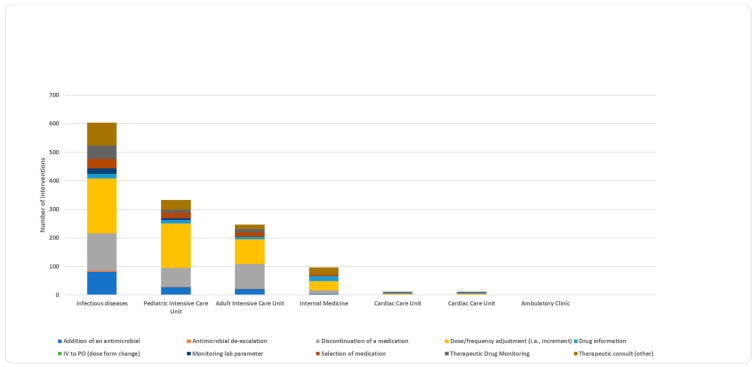
Types of interventions across various units in hospitals.

Although dose or frequency adjustment was the most common intervention in most hospital units (Figure 4 and Supplementary Table S5), some distinct patterns warrant attention. Therapeutic drug monitoring (TDM) accounted for the majority of interventions (41.7%) in cardiac care units (CCUs), while therapeutic consults made up over a quarter of the interventions in the internal medicine unit (27.1%) (Supplementary Table S5). Moreover, the percentage of medication discontinuation interventions was comparable to dose or frequency adjustment interventions in the adult ICU unit (34.8% for both).

There was a strong correlation between hospital units and intervention type (*p* = 0.001) (Supplementary Tables S6 and S7). Following a post hoc analysis to ascertain the precise location of the significant difference, four significant differences were identified (Table 3). In the adult ICU, the intervention (discontinue or de-escalate medication) was substantially more common than the other interventions (35.4%, *p* < 0.000) (Table 3). The drug information intervention was substantially more prevalent than other interventions in the internal medicine unit (44.8%, *p* < 0.000). Finally, in PICU, dose modification and monitoring were more prevalent than other interventions (51.7%, *p* < 0.000) (Table 3).

## 4. Discussion

Results from this long-term retrospective study provide additional evidence to support the value of clinical pharmacy services in general and in antimicrobial stewardship specifically. A growing body of evidence has explored the impact of pharmacy interns in diverse healthcare settings [14,15,16]. However, this study focuses explicitly on antimicrobial interventions during clinical rotations, providing unique insights into pharmacy interns’ role in infectious disease management.

This study demonstrated a significant impact of pharmacy interns on optimizing antimicrobial therapy, with a high number of interventions (1295) and a notable acceptance rate by physicians (91.6% directly implemented, 4.02% implemented with modifications). These findings suggest that physicians value pharmacy interns’ contributions. To the best of our knowledge, no prior research specifically addressed physician acceptance rate of pharmacy interns’ antimicrobial interventions. However, physicians’ acceptance rate reported in previous research of nonspecific interventions suggested by pharmacy interns was variable. A published study of pharmacy interns’ interventions in an ambulatory care setting in the United States (US) showed a 77% acceptance rate [17]. An acceptance rate of more than 80% was documented in another study in the US that evaluated three-year interventions of pharmacy students. However, in agreement with our study findings, other research showed a more than 90% acceptance rate [18].

The highest proportion of interventions was captured in a general hospital with one clinical preceptor (66% in hospital D) and in a specialized hospital that served two patient populations (25.7% in hospital A). Pharmacy interns were less likely to make interventions in a general hospital with eight clinical pharmacists (0.5%). Dose or frequency adjustments and medication discontinuations were the most common intervention types, with more than 85% of interventions implemented to increase efficacy (37.3%), reduce antimicrobial-related toxicities, and avoid unnecessary exposure to antimicrobial therapy. These interventions are important outcomes of antimicrobial stewardship programs [19]. Notably, there was a very low level of intravenous to oral switch interventions, underscoring a potential area for enhanced focus in future stewardship efforts. The current study is not designed to capture the economic outcomes of recorded interventions. Multiple studies conducted in various settings support the beneficial clinical and economic value of these recorded interventions [11,20]. A systematic review assessed the economic outcomes of ASP in a hospital setting and found that interventions aimed at optimizing antimicrobial use led to an average cost reduction of USD 732 per patient in US studies [21]. These savings were attributed to factors such as reduced antimicrobial consumption and shorter hospital stays.

Vancomycin, colistin, and meropenem were the most frequently intervened medications in the present research. These antimicrobials have complex dosing requirements and necessitate individualized therapy and close monitoring by clinical pharmacists [22,23]. The complexity of their pharmacokinetics and pharmacodynamics, coupled with the emergence of multidrug-resistant organisms, calls for customized dosing and vigilant monitoring. Additionally, acutely and critically ill patients often experience altered renal and/or hepatic functions, which necessitate individualized management to achieve optimal efficacy while minimizing adverse effects. Sepsis is the most common cause of acute kidney injury [24], with reported incidences ranging between 30% and 60% in intensive care patients [25]. Furthermore, higher doses or combinations of antimicrobials are typically needed to address antimicrobial resistance [26,27]. These factors collectively underscore the importance of appropriate dose or frequency adjustments. Due to their narrow therapeutic index, vancomycin and colistin necessitate meticulous monitoring and adjustments to ensure efficacy while preventing toxicity, such as nephrotoxicity [28,29]. Most interventions involving these antimicrobials fall under the category of TDM. Other studies have highlighted the significant impact of pharmacist-led TDM programs in enhancing medication safety and patient outcomes in critical care settings [30]. However, the most common intervention with ceftriaxone is medication discontinuation, possibly due to inappropriate use, aligning with other studies suggesting that over half of ceftriaxone prescriptions are inappropriate [31,32].

In terms of subclasses, glycopeptides had the most frequent interventions, followed by cephalosporins, which have a preferred safety profile. However, one of the challenges associated with cephalosporins is their dosing complexity, attributed to several factors such as infection site and severity, patient’s age, body weight, and kidney function.

The current study also highlighted context-specific intervention patterns encountered in different hospital units, emphasizing the dynamic nature of clinical pharmacy practice due to the variation in patient needs across diverse training sites. The emphasis on discontinuing and de-escalating antimicrobials is more prominent in intensive care rotations. Initially, broad-spectrum antimicrobials and their combinations are expected to be used for critical cases, often prolonged without de-escalation or discontinuation [33]. However, strategies for de-escalation or discontinuation have shown better clinical outcomes, underscoring the crucial role of pharmacy interns’ recommendations in intensive care settings [34,35]. Conversely, the emphasis on therapeutic consults in internal medicine rotation reflects the diverse medical conditions that pharmacy interns encounter. This finding aligns with other studies that have highlighted the substantial contributions of pharmacists in providing medication-related consultations in internal medicine settings [36,37].

These context-specific intervention patterns underscore the importance of tailored approaches to pharmacy practice, where interventions are guided by the unique clinical needs and treatment priorities of each hospital unit. By recognizing and addressing these differences, pharmacy interns can effectively contribute to patient care and medication safety across a diverse range of clinical settings. Moreover, these findings highlight the importance of interdisciplinary collaboration, where pharmacists work closely with physicians, nurses, and other healthcare professionals to optimize patient outcomes and enhance the quality of care delivery.

The findings in this report are subject to several limitations that warrant consideration. Notably, the retrospective review of interventions might limit the accuracy and depth of the collected data. The interventions were self-reported by pharmacy interns, potentially introducing reporting bias. All interventions were reviewed and approved by KKU clinical preceptors before being communicated to physicians, which likely contributed to the high acceptance rates observed. Unfortunately, we cannot determine if the preceptors’ approval had any impact on physicians’ acceptance rate. Furthermore, the study lacked the ability to ascertain patient-care outcomes, such as avoiding hospitalization, shortening length of stay, or enhancing patient quality of life. Additionally, evaluating economic outcomes and satisfaction levels among patients and providers were also outside the scope of this study. The generalizability of the findings to other healthcare settings or populations may be limited, and further research is needed to explore intervention patterns and their clinical and economic outcomes. Additionally, the impact of interventions on patient outcomes and economic benefits, though implied, requires more direct investigation.

## 5. Conclusions

Involving pharmacy interns in antimicrobial-related activities under clinical preceptors’ supervision was effective, as evidenced by the high acceptance rate of interns’ recommendations by physicians. Utilizing pharmacy interns for supervised antimicrobial-related activities may help alleviate the shortage of clinical pharmacists in certain regions to support the Saudi National Action Plan for Combating Antibiotic-resistant Bacteria. Future studies could focus on evaluating the long-term clinical and economic impacts of pharmacy interns’ interventions.

## Figures and Tables

**Table 1:** Characteristics of pharmacy interns’ interventions from 2017 to 2022 (*n* = 1295).

Clinical Intervention Variables	*n* (%)
Hospitals	
Specialized hospital A (two patient populations)	333 (25.7%)
Specialized hospital B (one patient population)	2 (0.2%)
General hospital C	6 (0.5%)
General hospital D	855 (66.0%)
General hospital E	99 (7.6%)
Hospital units	
Infectious diseases	604 (46.6%)
Pediatric intensive care unit	333 (25.7%)
Adult intensive care unit	247 (19.1%)
Internal medicine	96 (7.4%)
Cardiac care unit	12 (0.9%)
Psychiatry	2 (0.2%)
Ambulatory clinic	1 (0.1%)
Type of interventions	
Dose/frequency adjustment	470 (36.3%)
Discontinuation	296 (22.9%)
Therapeutic consult	159 (12.3%)
Addition of antimicrobial	134 (10.3%)
Therapeutic drug monitoring	73 (5.6%)
Selection of medication	71 (5.5%)
Drug information	52 (4.0%)
Monitoring lab parameter	29 (2.2%)
De-escalation	7 (0.5%)
Switching from intravenous (IV) to oral (PO)	4 (0.3%)
Clinical significance	
Increasing the efficacy of antimicrobial therapy	483 (37.3%)
Reducing adverse effects/toxicities related to antimicrobial therapy	347 (26.8%)
Avoiding unnecessary exposure to antimicrobial therapy	282 (21.8%)
Unknown significance	183 (14.1%)
Physician response to intervention	
Accepted	1186 (91.6%)
Accepted with modification	52 (4.0%)
Rejected	57 (4.4%)

**Table 2:** Drug classes to which the interventions were applied, ordered by the number of interventions (*n* = 1295).

Drug Classes *n* (%)	
Antibacterial agents	1130 (87.3)
Antifungal agents	77 (5.9)
Antiviral agents	63 (4.9)
Multiple classes	20 (1.5)
Antimalarial agents	4 (0.3)
Anthelmintics	1 (0.1)

**Table 3:** Types of intervention in various hospital units.

Intervention Type	ID	Adult ICU	Internal Medicine	PICU
Select/add medication	19.0% (0.0051)	14.2% (0.36812)	8.3% (0.0357)	14.1% (0.2713)
Discontinue/de-escalate medication	22.4% (0.3173)	**35.6% (0.0000)**	12.5% (0.0069)	20.1% (0.0891)
Drug information	15.7% (0.6171)	**9.3% (0.0009)**	**44.8% (0.0000)**	14.1% (0.2301)
Dose modification and monitoring	42.9% (0.3681)	40.9% (0.2713)	34.4% (0.0455)	**51.7% (0.0014)**

The table displays the percentages and corresponding *p*-values. The bold font indicates the significant *p*-value after applying the Bonferroni correction. Post hoc analysis was conducted for the previous chi-square test with a Bonferroni correction that adjusts the significance level of the *p*-value (a *p*-value is considered significant if it is less than 0.003125, calculated as 0.05 divided by 16).

## Data Availability

The original contributions presented in the study are included in the article/Supplementary Material, further inquiries can be directed to the corresponding author.

## Supplementary Materials

The following supporting information can be downloaded at: https://www.mdpi.com/article/10.3390/jcm13175060/s1, Table S1: Common types of interventions across various drug classes; Table S2: Drug classes and subclasses to which the interventions were applied (from higher to lower); Table S3: Drug classes and subclasses to which the interventions were applied; Table S4: Drugs to which the interventions were applied (from higher to lower) (*n* = 1295); Table S5: Common types of interventions across various hospital units; Table S6: Differences in the types of interventions among hospital units; Table S7: Chi-square test to examine the differences in types of interventions among hospital units.

## References

[1] Murray C.J., Ikuta K.S., Sharara F., Swetschinski L., Aguilar G.R., Gray A., Han C., Bisignano C., Rao P., Wool E. (2022). Global burden of bacterial antimicrobial resistance in 2019: A systematic analysis. Lancet.

[2] Wallis R.S., O’Garra A., Sher A., Wack A. (2023). Host-directed immunotherapy of viral and bacterial infections: Past, present and future. Nat. Rev. Immunol..

[3] Llor C., Bjerrum L. (2014). Antimicrobial resistance: Risk associated with antibiotic overuse and initiatives to reduce the problem. Ther. Adv. Drug Saf..

[4] Thompson T. (2022). The staggering death toll of drug-resistant bacteria. Nature.

[5] World Health Organization Antimicrobial Resistance. https://www.who.int/news-room/fact-sheets/detail/antimicrobial-resistance.

[6] Karanika S., Paudel S., Grigoras C., Kalbasi A., Mylonakis E. (2016). Systematic Review and Meta-analysis of Clinical and Economic Outcomes from the Implementation of Hospital-Based Antimicrobial Stewardship Programs. Antimicrob. Agents Chemother..

[7] Davey P., Marwick C.A., Scott C.L., Charani E., McNeil K., Brown E., Gould I.M., Ramsay C.R., Michie S. (2017). Interventions to improve antibiotic prescribing practices for hospital inpatients. Cochrane Database Syst. Rev..

[8] Monmaturapoj T., Scott J., Smith P., Abutheraa N., Watson M.C. (2021). Pharmacist-led education-based antimicrobial stewardship interventions and their effect on antimicrobial use in hospital inpatients: A systematic review and narrative synthesis. J. Hosp. Infect..

[9] Kuo G.M., Touchette D.R., Marinac J.S. (2013). Drug errors and related interventions reported by United States clinical pharmacists: The American College of Clinical Pharmacy practice-based research network medication error detection, amelioration and prevention study. Pharmacotherapy.

[10] Mas-Morey P., Ballesteros-Fernández A., Sanmartin-Mestre E., Valle M. (2018). Impact of clinical pharmacist intervention on antimicrobial use in a small 164-bed hospital. Eur. J. Hosp. Pharm..

[11] Salman B., Al-Hashar A., Al-Khirbash A., Al-Zakwani I. (2021). Clinical and Cost Implications of Clinical Pharmacist Interventions on Antimicrobial Use at Sultan Qaboos University Hospital in Oman. Int. J. Infect. Dis..

[12] Garau J., Bassetti M. (2018). Role of pharmacists in antimicrobial stewardship programmes. Int. J. Clin. Pharm..

[13] Nhan D., Lentz E.J.M., Steinberg M., Bell C.M., Morris A.M. (2019). Structure of Antimicrobial Stewardship Programs in Leading US Hospitals: Findings of a Nationwide Survey. Open Forum Infect. Dis..

[14] Abdulghani K.H., Aseeri M.A., Mahmoud A., Abulezz R. (2018). The impact of pharmacist-led medication reconciliation during admission at tertiary care hospital. Int. J. Clin. Pharm..

[15] Choi Y.J., Kim H. (2019). Effect of pharmacy-led medication reconciliation in emergency departments: A systematic review and meta-analysis. J. Clin. Pharm. Ther..

[16] Alghamdi A., Alhulaylah F., Al-Qahtani F., Alsallal D., Alshabanat N., Alanazi H., Alshehri G. (2022). Evaluation of pharmacy intern-led transition of care service at an academic hospital in Saudi Arabia: A prospective pilot study. Saudi Pharm. J..

[17] Shogbon A.O., Lundquist L.M. (2014). Student pharmacists’ clinical interventions in advanced pharmacy practice experiences at a community nonteaching hospital. Am. J. Pharm. Educ..

[18] Abdelhalim D., Mohundro B.L., Evans J.D. (2011). Role of student pharmacists in the identification and prevention of medication-related problems. J. Am. Pharm. Assoc..

[19] Zay Ya K., Win P.T.N., Bielicki J., Lambiris M., Fink G. (2023). Association Between Antimicrobial Stewardship Programs and Antibiotic Use Globally: A Systematic Review and Meta-Analysis. JAMA Netw. Open.

[20] Leache L., Aquerreta I., Aldaz A., Monedero P., Idoate A., Ortega A. (2020). Clinical and economic impact of clinical pharmacist interventions regarding antimicrobials on critically ill patients. Res. Social. Adm. Pharm..

[21] Nathwani D., Varghese D., Stephens J., Ansari W., Martin S., Charbonneau C. (2019). Value of hospital antimicrobial stewardship programs [ASPs]: A systematic review. Antimicrob. Resist. Infect. Control.

[22] Murphy M., Girdwood S.T., Scheetz M.H. (2020). Clinical Guideline Highlights for the Hospitalist: Therapeutic Monitoring of Vancomycin. J. Hosp. Med..

[23] Wang C., Bai C., Chen K., Du Q., Cheng S., Zeng X., Wang Y., Dong Y. (2024). International guidelines for the treatment of carbapenem-resistant Gram-negative Bacilli infections: A comparison and evaluation. Int. J. Antimicrob. Agents.

[24] Eswarappa M., Gireesh M.S., Ravi V., Kumar D., Dev G. (2014). Spectrum of acute kidney injury in critically ill patients: A single center study from South India. Indian J. Nephrol..

[25] Hoste E.A.J., Kellum J.A., Selby N.M., Zarbock A., Palevsky P.M., Bagshaw S.M., Goldstein S.L., Cerdá J., Chawla L.S. (2018). Global epidemiology and outcomes of acute kidney injury. Nat. Rev. Nephrol..

[26] Day T., Read A.F. (2016). Does High-Dose Antimicrobial Chemotherapy Prevent the Evolution of Resistance?. PLoS Comput. Biol..

[27] Coates A.R.M., Hu Y., Holt J., Yeh P. (2020). Antibiotic combination therapy against resistant bacterial infections: Synergy, rejuvenation and resistance reduction. Expert Rev. Anti Infect. Ther..

[28] Sanabria J., Garzón V., Pacheco T., Avila M.-P., Garcia J.-C., Jaimes D., Torres A., Bustos R.-H., Escobar-Perez J., Abril D. (2021). Estimation of the Difference in Colistin Plasma Levels in Critically Ill Patients with Favorable or Unfavorable Clinical Outcomes. Pharmaceutics.

[29] He N., Su S., Ye Z., Du G., He B., Li D., Liu Y., Yang K., Zhang X., Zhang Y. (2020). Evidence-based guideline for therapeutic drug monitoring of vancomycin: 2020 update by the division of therapeutic drug monitoring, Chinese pharmacological society. Clin. Infect. Dis..

[30] Jantarathaneewat K., Phodha T., Singhasenee K., Katawethiwong P., Suwantarat N., Camins B., Wongphan T., Rutjanawech S., Apisarnthanarak A. (2023). Impact of pharmacist-led multidisciplinary team to attain targeted vancomycin area under the curved monitoring in a tertiary care center in Thailand. Antibiotics.

[31] Berhe Y.H., Amaha N.D., Ghebrenegus A.S. (2019). Evaluation of ceftriaxone use in the medical ward of Halibet National Referral and teaching hospital in 2017 in Asmara, Eritrea: A cross sectional retrospective study. BMC Infect. Dis..

[32] Delorme C., Viel-Thériault I., Moradipour T., Le Saux N. (2020). Drug use evaluation (DUE) of ceftriaxone: A quality metric in a pediatric hospital. Off. J. Assoc. Med. Microbiol. Infect. Dis. Can..

[33] Zhao K., Zhang Z., Liang Y., Wang Y., Cai Y. (2023). Effect of antimicrobial de-escalation strategy on 14-day mortality among intensive care unit patients: A retrospective propensity score-matched cohort study with inverse probability-of-treatment weighting. BMC Infect. Dis..

[34] Hughes J., Huo X., Falk L., Hurford A., Lan K., Coburn B., Morris A., Wu J. (2017). Benefits and unintended consequences of antimicrobial de-escalation: Implications for stewardship programs. PLoS ONE.

[35] Hamilton W.L., Pires S.M., Lippett S., Gudka V., Cross E.L.A., Llewelyn M.J. (2020). The impact of diagnostic microbiology on de-escalation of antimicrobial therapy in hospitalised adults. BMC Infect. Dis..

[36] Rahayu S.A., Widianto S., Defi I.R., Abdulah R. (2021). Role of Pharmacists in the Interprofessional Care Team for Patients with Chronic Diseases. J. Multidiscip. Healthc..

[37] Firkus D., McCoy R.G., Matulis J., Kessler M., Mara K., Herges J. (2023). Evaluation of pharmacist consults within a collaborative enhanced primary care team model to improve diabetes care. PLoS ONE.

