# Supplementary material for: Evaluation of Pharmacy Intern Interventions on Antimicrobial Use in University-Affiliated Hospitals: A Retrospective Analysis
Source: J Clin Med. 2024 Aug 26;13(17):5060. doi: 10.3390/jcm13175060 (PMC11395848; doi:10.3390/jcm13175060)
Supplement: Supplementary file 1 [file jcm-13-05060-s001.zip › jcm-3121547-supplementary.pdf]

Journal of Clinical Medicine

Table S1: Common types of interventions across various drug classes

Drug/intervention	Addition of an antimicrobial	Antimicrobial de-escalation	Discontinuation of a medication	Dose/frequency adjustment (i.e., increment)	Drug information	IV to PO (dose form change)	Monitoring lab parameter	Selection of medication	TDM	Therapeutic consult (other)	Total
Anthelmintics	0 (0)	0 (0)	0 (0)	1 (100)	0 (0)	0 (0)	0 (0)	0 (0)	0 (0)	0 (0)	1 (100)
Antibacterial agents	118 (10.4)	6 (0.5)	269 (23.8)	419 (37.1)	37 (3.3)	4 (0.4)	24 (2.1)	58 (5.1)	73 (6.5)	122 (10.8)	1130 (100)
Antifungal agents	6 (7.8)	1 (1.3)	12 (15.6)	19 (24.7)	9 (11.7)	0 (0)	3 (3.9)	8 (10.4)	0 (0)	19 (24.7)	77 (100)
Antimalarial agents	0 (0)	0 (0)	0 (0)	2 (50)	0 (0)	0 (0)	0 (0)	0 (0)	0 (0)	2 (50)	4 (100)
Antiviral agents	7 (11.1)	0 (0)	13 (20.6)	29 (46)	5 (7.9)	0 (0)	2 (3.2)	0 (0)	0 (0)	7 (11.1)	63 (100)
Multiple classes	3 (15)	0 (0)	2 (10)	0 (0)	1 (5)	0 (0)	0 (0)	5 (25)	0 (0)	9 (45)	20 (100)

Data presented as n (%)

Table S2: Drug classes and subclasses to which the interventions were applied (from higher to lower)

Antibacterial agents (n= 1130) (%)	Antifungal agents (n=77) (%)	Antiviral agents (n=63) (%)	Multiple classes (n=20) (%)	Antimalarial agents (n=4) (%)	Anthelmintics (n=1) (%)
Glycopeptides (23.4)	Polyenes (41.6)	Neuraminidase inhibitors (44.4)		Artemisinins (75)	Benzimidazoles (100)
Cephalosporins (13.6)	Azoles (36.4)	Purine nucleosides (41.3)		Quinolines (25)	
Carbapenems (11.2)	Echinocandins (20.8)	Integrase strand transfer inhibitors (6.3)			
Polypeptide (9.3)	Unspecified (1.3)	Multiple classes (6.3)			
Unspecified (5.8)		NRTIs (1.6)			
Penicillin/ β- lactamase inhibitors. (5.6)					
Fluoroquinolones (4.6)					
Aminoglycosides (4.5)					
Nitroimidazole (4.2)					
Sulfonamides (3.6)					
Macrolides (3.2)					
Penicillin (2.9)					
Oxazolidinones (2.7)					
Glycylcycline (1.9)					
Rifamycin (1)					
Lincosamide (0.9)					
Monobactams (0.4)					
Hydrazide derivatives (0.4)					
Cyclic lipopeptides (0.2)					
Ethylenediamine derivatives (0.2)					
Pyrazines (0.2)					
Tetracyclines (0.2)					
Multiple classes (0.1)					

Table S3: Drug classes and subclasses to which the interventions were applied

Class	Subclass	Addition of an antimicrobial	Antimicrobial de-escalation	Discontinuation of a medication	Dose/frequency adjustment (i.e., increment)	Drug information	IV to PO (dose form change)	Monitoring lab parameter	Selection of medication	TDM	Therapeutic consult (other)	Total
Anthelmintics	Benzimidazoles	0	0	0	1	0	0	0	0	0	0	1
	Total	0	0	0	1	0	0	0	0	0	0	1
Antibacterial agents	Aminoglycosides	5	0	14	18	2	0	2	0	4	6	51
	Carbapenems	12	1	28	68	4	0	1	4	0	9	127
	Cephalosporins	10	2	47	64	8	1	1	12	0	9	154
	Cyclic lipopeptides	1	0	0	0	0	0	1	0	0	0	2
	Ethylenediamine derivatives	0	0	0	2	0	0	0	0	0	0	2
	Fluoroquinolones	7	0	21	14	1	1	1	3	0	4	52
	Glycopeptides	15	1	41	103	6	0	4	11	69	14	264
	Glycylcycline	8	0	7	2	0	0	2	1	0	1	21
	Hydrazide derivatives	1	0	0	0	0	0	1	0	0	2	4
	Lincosamides	2	0	1	4	1	0	0	2	0	0	10
	Macrolides	7	0	11	6	0	0	0	3	0	9	36
	Monobactams	2	0	1	1	1	0	0	0	0	0	5
	Multiple classes	0	0	0	0	0	0	1	0	0	0	1
	Nitroimidazole	10	0	28	5	0	2	0	2	0	1	48
	Oxazolidinones	3	0	8	2	2	0	4	3	0	8	30
	Penicillin	6	0	4	20	2	0	0	0	0	1	33
	Penicillin/ β-lactamase inhibitors	2	1	27	20	3	0	0	6	0	4	63
	Polypeptide	20	0	18	53	3	0	0	7	0	4	105

	Pyrazines	0	0	0	2	0	0	0	0	0	0	2
	Rifamycin	2	0	2	1	1	0	1	1	0	3	11
	Sulfonamides	5	0	5	29	1	0	0	1	0	0	41
	Tetracyclines	0	0	1	1	0	0	0	0	0	0	2
	Unspecified	0	1	5	4	2	0	5	2	0	47	66
	Total	118	6	269	419	37	4	24	58	73	122	1130
Antifungal agents	Azoles	2	0	1	13	1	0	3	1	0	7	28
	Echinocandins	2	0	3	1	5	0	0	2	0	3	16
	Polyenes	2	1	8	5	3	0	0	5	0	8	32
	Unspecified	0	0	0	0	0	0	0	0	0	1	1
	Total	6	1	12	19	9	0	3	8	0	19	77
Antimalarial agents	Artemisinin	0	0	0	2	0	0	0	0	0	1	3
	Quinolines	0	0	0	0	0	0	0	0	0	1	1
	Total	0	0	0	2	0	0	0	0	0	2	4
Antiviral agents	Integrase strand transfer inhibitors	0	0	0	2	1	0	0	0	0	1	4
	Multiple classes	0	0	0	0	1	0	2	0	0	1	4
	Neuraminidase inhibitors	6	0	10	10	1	0	0	0	0	1	28
	NRTIs	0	0	0	1	0	0	0	0	0	0	1
	Purine nucleosides	1	0	3	16	2	0	0	0	0	4	26
	Total	7	0	13	29	5	0	2	0	0	7	63
Multiple classes	Multiple classes	3	0	2	0	1	0	0	5	0	9	20
	Total	3	0	2	0	1	0	0	5	0	9	20

Table S4: Drugs to which the interventions were applied (from higher to lower) (n= 1,295)

Drug n (%)			
Vancomycin	269 (20.8)	Aztreonam	5 (0.4)
Colistin	105 (8.1)	Clarithromycin	5 (0.4)
Meropenem	99 (7.6)	Moxifloxacin	5 (0.4)
Unspecified antibacterial	66 (5.1)	Caspofungin	4 (0.3)
Piperacillin/Tazobactam	65 (5)	Elvitegravir/cobicistat/emtricitabine/tenofovir	4 (0.3)
Ceftriaxone	49 (3.8)	Isoniazid	4 (0.3)
Metronidazole	48 (3.7)	Unspecified aminoglycosides	3 (0.2)
Trimethoprim/Sulfamethoxazole	41 (3.2)	Artesunate	3 (0.2)
Ceftazidime	35 (2.7)	Itraconazole	3 (0.2)
Linezolid	32 (2.5)	Nystatin	3 (0.2)
Levofloxacin	31 (2.4)	Raltegravir	3 (0.2)
Amphotericin B	30 (2.3)	Daptomycin	2 (0.2)
Oseltamivir	29 (2.2)	Ethambutol	2 (0.2)
Acyclovir	26 (2)	Pyrazinamide	2 (0.2)
Gentamicin	26 (2)	Teicoplanin	2 (0.2)
Azithromycin	24 (1.9)	Voriconazole	2 (0.2)
Ampicillin	23 (1.8)	Albendazole	1 (0.1)
Fluconazole	23 (1.8)	Amoxicillin	1 (0.1)
Imipenem/Cilastatin	23 (1.8)	Ampicillin/Sulbactam	1 (0.1)
Amikacin	22 (1.7)	Cefixime	1 (0.1)
Tigecycline	22 (1.7)	Cloxacillin	1 (0.1)
Ceftazidime/Avibactam	20 (1.5)	Dolutegravir	1 (0.1)
Cefotaxime	18 (1.4)	Doxycycline	1 (0.1)
Cefazolin	15 (1.2)	Entecavir	1 (0.1)
Ciprofloxacin	15 (1.2)	Fidaxomicin	1 (0.1)
Anidulafungin	12 (0.9)	Flucloxacillin	1 (0.1)
Rifampin	11 (0.8)	Fluoroquinolones	1 (0.1)
Cefuroxime	10 (0.8)	Hydroxychloroquine	1 (0.1)
Clindamycin	10 (0.8)	Rifampicin/Isoniazid	1 (0.1)
Cefepime	9 (0.7)	Rifaximin	1 (0.1)
Amoxicillin/Clavulanate	7 (0.5)	Unspecified tetracycline	1 (0.1)
Erythromycin	6 (0.5)	Unspecified antifungal	1 (0.1)
Unspecified Carbapenems	6 (0.5)		

Table S5: Common types of interventions across various hospital units

	ID	Ambulatory Clinic	CCU	Adult ICU	Internal Medicine	PICU	Psychiatry
Addition of an antimicrobial	81 (13.4)	0 (0)	0 (0)	21 (8.5)	4 (4.2)	28 (8.4)	0 (0)
Antimicrobial de-escalation	4 (0.7)	0 (0)	0 (0)	2 (0.8)	0 (0)	1 (0.3)	0 (0)
Discontinuation of a medication	131 (21.7)	0 (0)	1 (8.3)	**86 (34.8)**	12 (12.5)	66 (19.8)	0 (0)
Dose/frequency adjustment (i.e., increment)	**192 (31.8)**	0 (0)	3 (25)	**86 (34.8)**	**32 (33.3)**	**155 (46.5)**	**2 (100)**
Drug information	14 (2.3)	0 (0)	2 (16.7)	7 (2.8)	17 (17.7)	12 (3.6)	0 (0)
IV to PO (dose form change)	2 (0.3)	0 (0)	0 (0)	1 (0.4)	0 (0)	1 (0.3)	0 (0)
Monitoring lab parameter	19 (3.1)	0 (0)	1 (8.3)	2 (0.8)	1 (1)	6 (1.8)	0 (0)
Selection of medication	34 (5.6)	0 (0)	0 (0)	14 (5.7)	4 (4.2)	19 (5.7)	0 (0)
TDM	46 (7.6)	0 (0)	**5 (41.7)**	12 (4.9)	0 (0)	10 (3)	0 (0)
Therapeutic consult (other)	81 (13.4)	**1 (100)**	0 (0)	16 (6.5)	26 (27.1)	35 (10.5)	0 (0)
All	604 (100)	1 (100)	12 (100)	247 (100)	96 (100)	333 (100)	2 (100)

ID: Infectious Disease; CCU: Cardiac Care Unit; ICU: Intensive Care Unit; PICU: Pediatric Intensive Care Unit.

Table S6: Differences in the types of interventions among hospital units

Intervention/ Unit	ID	Adult ICU	Internal Medicine	PICU
Select/add medication	115 (19.04)	35 (14.17)	8 (8.33)	47 (14.11)
Discontinue/De-escalate medication	135 (22.35)	88 (35.63)	12 (12.5)	67 (20.12)
Drug information	95 (15.73)	23 (9.31)	**43 (44.79)**	47 (14.11)
Dose modification and monitoring	**259 (42.88)**	**101 (40.89)**	33 (34.38)	**172 (51.65)**
Total	604 (100)	247 (100)	96 (100)	333 (100)

Table S7: Chi-square test to examine the differences in types of interventions among hospital units

Chi-Square Tests			
	Value	df	Asymptotic Significance (2-sided)
Pearson Chi-Square	94.147a	9	<.001
Likelihood Ratio	79.462	9	<.001
Linear-by-Linear Association	6.425	1	0.011
N of Valid Cases	1280		
